# Cytosine methylation and hydroxymethylation mark DNA for elimination in *Oxytricha trifallax*

**DOI:** 10.1186/1756-8935-6-S1-P8

**Published:** 2013-03-18

**Authors:** John R Bracht, David H Perlman, Laura F Landweber

**Affiliations:** 1 Ecology & Evolutionary Biology Department, Princeton University Washington Rd. Princeton, NJ 08544, USA; 2 Collaborative Proteomics and Mass Spectrometry Center, Molecular Biology Department and the Lewis-Sigler Institute for Integrative Genomics, Princeton University, Washington Rd., Princeton, NJ, 08544, USA

## 

Cytosine methylation of DNA is conserved across eukaryotes and plays important functional roles regulating gene expression during differentiation and development in animals, plants and fungi. Hydroxymethylation was recently identified as another epi-genetic modification marking genes important for pluripotency in embryonic stem cells. Here we describe *de novo* cytosine methylation and hydroxymethylation in the ciliate *Oxytricha trifallax*. These DNA modifications occur only during nuclear development and programmed genome rearrangement. We describe these modifications in three classes of eliminated DNA: germline-limited transposons and satellite repeats, aberrant DNA rearrangements, and DNA from the parental genome undergoing degradation. Methylation and hydroxymethylation generally occur on the same sequence elements, modifying cytosines in all sequence contexts. We show that the DNA methyltransferase-inhibiting drugs azacitidine and decitabine induce demethylation of both somatic and germline sequence elements during genome rearrangements, with consequent elevated levels of germline-limited repetitive elements in exconjugant cells. These data strongly support a functional link between cytosine DNA methylation/hydroxymethylation and genome stability, highlighting the ability of the cells to distinguish and specifically methylate aberrant chromosomes but not their properly structured isoforms. In addition, we identify a motif strongly enriched in some methylated/hydroxymethylated regions of parental MAC chromosomes, and we propose that this motif recruits DNA methylation machinery to specific chromosomal regions in the parental macronucleus. No recognizable methyltransferase enzyme has yet been described in *O. trifallax*, raising the possibility that it might employ a novel cytosine methylation machinery to mark DNA sequences for elimination during genome rearrangements.

